# Optimization design of brushless DC motor based on improved JAYA algorithm

**DOI:** 10.1038/s41598-024-54582-z

**Published:** 2024-03-05

**Authors:** Yuan Cheng, Xueli Lyu, Shasha Mao

**Affiliations:** School of Control Engineering, Wuxi Institute of Technology, Wuxi, 214121 China

**Keywords:** JAYA algorithm, Brushless DC motor, Optimization problems, Mathematical model, Parameter optimization, Electrical and electronic engineering, Mechanical engineering

## Abstract

Brushless direct current motor is widely used in industrial production because of its simple structure, wide speed range and low noise. To improve the operation efficiency of brushless DC motor and reduce the production and application costs, the optimization of brushless DC motor is analyzed by introducing the JAYA algorithm. This method determines the optimal parameters of a brushless DC motor using the theory of electromagnetic structure parameter selection and efficiency calculation. The population diversity of the JAYA algorithm is improved through an empirical learning strategy, and an adaptive strategy is introduced to balance the development ability and search performance of the algorithm. This ensures population diversity and improves convergence speed. The experiment showcases that the improved JAYA algorithm has a lower rank average in unimodal function operations, demonstrating stronger local development ability and better stability. It exhibits strong search ability in many local optima of multimodal functions. Moreover, the motor's average efficiency after optimization is 94.48%. The algorithm reaches the global optimum after approximately 40 iterations and offers faster convergence speed and higher accuracy. The adaptive JAYA algorithm is stable at around 93% when the number of iterations reaches 90, with a maximum efficiency of 95.3%. It is 5–12 percentage points higher than the other three comparison algorithms. The optimal solution of the motor parameters in the adaptive JAYA algorithm is closest to the theoretical parameter optimization value, meeting both the constraints of variables and the constraints of the model. The stator diameter, tooth magnetic induction, winding current density, air gap magnetic induction, and stator yoke magnetic induction values are 201.5 mm, 1.8 T, 2.049 A/mm^2^, 0.63 T, and 0.91 T, respectively. The research overcomes the problem of parameter optimization in the optimization design of brushless DC motor, improving their economic value of brushless DC motor in industrial production and application.

## Introduction

Thanks to the boost of the social economy, technology has also made progress, and the research field related to motors is also constantly innovating and improving. As one of the common types of motors, the joint development of electronic technology, material science, automatic control theory, and microelectronics science has also driven the development of brushless DC motors, especially the optimization problem (OPR) of brushless DC motors, which has become a research hotspot^1^. Motor optimization design typically involves creating a mathematical model that meets specific motor design standards, user requirements and certain constraints. By optimizing the relevant parameters or structure of the motor, the efficiency and some performance indexes of the motor can achieve the optimal effect. The design analysis is to preset all kinds of parameters of the motor, and then calculate all kinds of motor performance indicators by calculation method according to the motor model. The calculated indicators are compared with the performance indicators required by the design. If they are not up to standard, the parameters of the motor will continue to be changed, and the cycle calculation indicators will reach the final requirements. The traditional solution to OPR entails creating a mathematical model on the ground of the optimization objective and searching for the optimal solution through numerical or analytical computing methods, as well as direct search methods^2^. However, traditional solving methods have problems such as high computational complexity and long computational time, with the resolution process heavily dependent on initial values^3^. Recently, with the rise of intelligent optimization algorithms, motor OPR have gradually introduced algorithms with simple structures and easy parallel processing, among which the JAYA algorithm is the mainstream. The performance optimization of common bio-inspired algorithms is greatly influenced by parameter settings. Unreasonable parameter settings can directly impact the optimization results. At the same time, the parameter selection of these optimization algorithms is strongly correlated with the problem being solved, and parameter values may need to be adjusted for new optimization problems. The relevant studies often utilized the JAYA algorithm to the relevant OPR, which can accelerate the convergence speed (CSP) of the population through a single information exchange. Individual updates are only guided by the best and worst solutions^4^. However, the JAVA algorithm also suffers from a single way of information exchange, which can easily lead to a lack of population diversity and premature convergence, leading to local optima. Therefore, based on this background, the study proposed an improved JAYA algorithm to optimize key parameters such as stator diameter, tooth magnetic induction, and winding current density for brushless DC motors. The algorithm proposed in the study maintains high stability in optimizing key parameters of the motor, effectively avoiding local optima and improving convergence rate. This study can help improve the operational efficiency of motors and promote further advancements in brushless DC motor technology. The innovations of the research are as follows: Firstly, it solves the parameter optimization problem of brushless DC motor using an improved JAYA algorithm. Secondly, it enhances the population diversity of the JAYA algorithm through an empirical learning strategy. Thirdly, it introduces an adaptive strategy to balance the development ability and search performance of the JAYA algorithm, ensuring population diversity and improving CSP. The main contributions of the research are as follows.

1. An improved JAYA algorithm is proposed. Based on the JAYA algorithm, this algorithm proposes an empirical learning strategy, which improves the diversity of the algorithm population by making full use of the experience of other individuals to learn, while avoiding the algorithm falling into local optimization. The experimental results show that the algorithm is competitive in solving the optimal design problem for brushless DC motor.
2. An adaptive JAYA algorithm is proposed. The algorithm introduces adaptive selection mechanism to adaptively select different learning strategies and balance the exploration and development ability of the algorithm. An adaptive weight strategy is introduced into the learning strategy of basic JAYA algorithm to control the degree that each individual approaches the best solution and avoids the worst solution. On this basis, a hybrid learning strategy based on the optimal solution and other individual experiences is proposed to maintain the diversity of the algorithm population and improve the local search ability of the algorithm. The chaotic elite search strategy is introduced to refine the optimal solution in each generation. The experimental results show that the proposed algorithm h is highly competitive in terms of global optimization, convergence rate and algorithm stability. Additionally, it enhances motor operation efficiency.
3. The performance and universality of the algorithm is tested. The proposed algorithm is very competitive in solving some complex test functions, exhibiting high precision and convergence. Additionally, it displays good universality in other problems.

This study consists of four. The first provides an overview of the OPR of brushless DC motors and the current research status of the JAVA algorithm. In the second part, based on the analysis of the mathematical optimization model of a brushless DC motor, the JAYA algorithm is put forward according to the characteristics of the optimization problem of a brushless DC motor, and it is applied to the optimization of stator diameter, tooth magnetic induction, winding current density, air gap magnetic induction and stator yoke magnetic induction parameters of brushless DC motor. The third analyzes the performance testing results and comparative experimental results of the improved JAYA algorithm. The fourth summarizes the conclusions of this study, at the same time, the shortcomings of the research are put forward.

## Related works

As one of the common types of motors, the OPR of brushless DC motors has been developed in recent years. Generally speaking, unexpected failures of sensorless brushless DC motors could lead to production downtime, expensive maintenance, and safety issues. Motor optimization technology has rapidly progressed, and some new and effective optimization methods are used in motor optimization problems. New optimization methods are mainly divided into two categories, namely model method and search method. The model method mainly establishes the loss model based on the motor parameters, selects the appropriate control quantity and solves its optimal solution. This allows for efficient optimization control with the help of other control quantities. The search method is more convenient, time-saving and labor-saving than the model method. The research reports of model method and search method analysis in electrode optimization design are as follows. K. Vanchinathan et al. proposed a work optimization technology strategy for speed control of sensorless brushless DC motors to address issues such as Hall effect sensors, Hall effect sensor misalignment, inverter open circuit fault diagnosis, and Hall effect sensor faults. This strategy adopted the bat algorithm, grey wolf optimization algorithm, and whale optimization algorithm for fault diagnosis of brushless DC motors. The numerical simulation results of Matlab/Simulink 2020a validated the strategy of simulating the above optimization technology on sensorless brushless DC motors. The relevant outcomes showcased that this method is very useful in sensorless brushless DC motor drive^5^. S. Hans et al. believed that an efficient speed controller is an important requirement for the operation of brushless DC motors. But adding an integral controller can cause load disturbances, control complexity, and some parameter changes. On the ground of this, the study proposed a plus integral controller to overcome the problems caused by adding an integral controller in brushless DC motors. In this architecture, the design of the controller included both a weight function and a transfer function. The controller was already in the brushless DC motor for detecting rotor position. The study introduced the particle swarm optimization (PSO) algorithm in this method help identify the optimal position of the rotor. The simulation results showed that the controller significantly reduced the torque obtained in the motor and improves speed, and the proposed technology provided better results than other existing controllers^6^.

In recent years, the rise of intelligent optimization algorithm has been widely concerned by scholars in the field of motor optimization, which have been used in search methods. Intelligent optimization algorithm is a new optimization algorithm, which is inspired by simulating natural phenomena or biological behavior characteristics through the combination of randomness and simple rules, and solves optimization problems in an efficient way. The design aims to balance fast CSP with the ability to escape local optima, enabling the search for the global optimum. K. Chakkarapani et al. used multi-objective optimization algorithms to design brushless DC motors. Its optimization objectives included maximizing output torque, minimizing volume, and minimizing total loss. Firstly, the study performed the sensitivity analysis to identify the maximum parameters that affect the performance of brushless DC motors. Subsequently, it used the performance metrics to select the optimal algorithm from Pareto Envelope Based Selection Algorithm (PESA), Pareto Archive Evolution Strategy (PAES), and Non Explicit Sorting Genetic Algorithm II (NSGA-II). Finally, the transient and thermal characteristics of brushless DC motors were studied using finite element method. The study compared the thermal results obtained from the above motors under different operating conditions with existing single objective optimization algorithms. The comparison demonstrated that the improved algorithm is significantly superior to existing single objective optimization algorithms^7^. Qin J et al. proposed a Worm algorithm-based parameter tuning approach for brushless DC motors to mitigate the challenges associated with adjusting control parameters like low control precision and limited applicability. Firstly, the study established a speed control model of a brushless DC motor with two-phase conduction and three-phase full bridge drive using the proportional integration method. Then, the study built fitness function of the controller using the integral absolute error. Finally, the study determined early optimization process, later motion rules, and peak extraction rules of WOA, and designed the controller parameter tuning process. The experiment illustrated that the whale algorithm has an average improvement of 2.56% in control performance at constant speed and 16.93% in sine speed. This method is useful for parameter adjustment in complex control, as it has higher control accuracy^8^.

The JAYA algorithm is a new meta heuristic algorithm with a simple structure. Zhang Y et al. proposed an enhanced JAYA algorithm to improve the global search ability. In this algorithm, local development was on the ground of defined upper and lower local attractors. The experiment demonstrated that the algorithm's strong capability to avoid local optima, as well as the effectiveness of the improvement strategy introduced by JAYA^9^. R. Rao et al. proposed an adaptive multi team disturbance guided JAYA algorithm that utilizes multiple teams for exploring the search space. Each team used the same population set, and each team had different perturbation or motion equations. Due to each team having different disturbance plans, the set of each team moving to a new location was unique. The study could update the motion equations of the teams with the worst performance on the ground of the advantages of the solutions generated by each team. It calculated the superiority of each team's solution on the ground of its fitness value and boundary violations. Compared with other common methods, the calculation test results showed that this method can significantly improve Jaya's CSP and the accuracy of the calculation results. This method was effective^10^. Bangyal W H and other scholars proposed an initialization technique for low difference sequences, which extends the performance of particle swarm optimization algorithms and makes them suitable for optimization problems. The practicality of the proposed solution was verified through single peak and multi peak benchmark tests, and the research has made a significant contribution to the quality of cost functions, integration, and diversity^11^. Bangyal W proposed an improved seasonal algorithm for solving optimization problems in current research, and applied it to numerical optimization solutions. The effectiveness of this method was verified through classical unimodal and multimodal benchmark functions^12^.

In summary, many experts have conducted research on the OPR of brushless DC motors and the application of JAVA algorithm in OPR processing. However, while experts may have an advantage in finding optimal solutions for optimization problems, the reliability and stability improvements are limited. The application of the JAYA algorithm in the optimal design of a brushless DC motor is still in the exploratory stage. In the optimization design of motor, the main problems are as follows. First, the global optimization problem. At present, the existing optimization methods cannot achieve the effect of finding the global optimum quickly and avoiding falling into the local optimum at the same time. However, motor optimization design is a complex engineering problem that exacerbates the difficulty of motor optimization. Secondly, the selection of motor parameters. Different motor problems require different optimization parameters, which can increase the difficulty of motor optimization. Thirdly, the optimal design method of motor. In addition to considering the electromagnetic scheme, the optimization design method should also consider the comprehensive multi-objective optimization problems of motor structure, noise and working temperature. By taking these factors into account, the accuracy of motor optimization can be further improved. Therefore, the research focuses on a the JAYA algorithm which can ensure the CSP and avoid premature convergence, and balance the development ability and search performance of the algorithm through an adaptive strategy. The aim is to provide technical support for optimizing brushless DC motors.

## Optimization of brushless DC motor based on JAYA algorithm

The optimal design of motor needs the design analysis and comprehensive design of motor. The optimal design of motor is a multi-constraint, multi-modal, multi-variable and complex mixed discrete optimization problem. In the problem of motor optimization, the selection of parameters such as stator diameter, magnetic induction in teeth, winding current density, magnetic induction in air gap and magnetic induction in stator yoke is very important to the optimization effect. Different optimization parameters will yield varying outcomes^13^. This study aims at improving the efficiency of brushless DC motor, transforming its parameter problem into optimization problem, and deducing the relationship between its internal parameters to build its optimization mathematical model. The performance optimization of bio-inspired algorithms, such as the Goose Bird Optimization Algorithm, Krill Swarm Algorithm, Bat Algorithm, Imperialist Competition Algorithm, Ant Colony Algorithm, Dove Swarm Algorithm and Backward Search Algorithm, has a great relationship with parameter setting, and unreasonable parameter setting will directly affect the optimization result of a brushless DC motor. At the same time, the parameter selection of this part of the optimization algorithm is strongly correlated with the problem itself, and the parameter values may be constantly adjusted for new optimization problems. Therefore, in the parameter optimization of a brushless DC motor, it is difficult for decision makers to set reasonable and effective parameters in a short time. This study analyzes the optimization of a brushless DC motor by introducing the JAYA algorithm. The optimization parameters of the motor are determined with the support of the selection of internal electromagnetic structure parameters and efficiency calculation theory. The population diversity of the JAYA algorithm is improved through an empirical learning strategy, and the adaptive strategy is introduced to balance the development ability and search performance of the JAYA algorithm. This ensures the population diversity and improves the CSP.

### Optimization of brushless DC motor on the ground of improved JAYA algorithm and parameter selection

**Figure 1:**
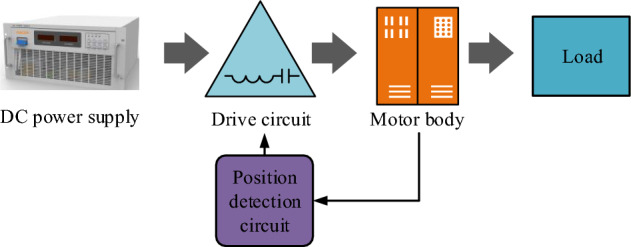
Basic structure of the motor.

For the optimization design problem of brushless DC motors, the research first introduces the structure and mathematical model of brushless DC motors. The body of a brushless DC motor includes a stator and a rotor. Figure 1 showcases the basic structure of the motor, indicating that the driving circuit and position detection circuit are responsible for the commutation task of the brushless DC motor. The electric winding of the motor is distributed on the stator, and the rotor is made of permanent magnet magnetic steel^14^.

The driving support source of brushless DC motor mainly relies on rotor rotation. When the position sensor of the motor detects the position signal of the rotor, the power switch connected to the commutation drive circuit and the armature winding controls the energization of the stator winding through conduction or shut-off operations. The upwardly advancing stator generates a rotating magnetic field that can propel the rotor to rotate. During the rotation of the rotor, the position sensor will continuously transmit signals, resulting in a change in the energized state of the armature. Within a conductor with the same magnetic pole, the direction of the current remains constant, generating a constant torque to support the rotation of the DC motor^15^. The inverter circuit structure of the motor contains six switch tubes, which adopts the conduction mode of a three-phase star full bridge inverter circuit (BIC) with "two two conduction". Table 1 shows the sequence control of the three-phase star type full BIC for the on–off of the switch tubes. The rotor of the motor rotates every 60 degrees within a cycle, and the sequence of energizing the stator winding and conducting the switch tubes will change. The motor can ensure the continuous rotation of the stator winding by controlling the switch tube's switching sequence.

**Table 1:** Switching tube on/off sequence.

Angle	60°	120°	180°	240°	300°	360°
Switching	1,4	1,6	3,6	3,2	5,2	5,4
A-phase	On	On	Off	On	On	Off
B-phase	On	Off	On	On	Off	On
C-phase	Off	On	On	Off	On	On

On the basis of clarifying the principle and structure of brushless DC motors, this study deduces the parameter relationships that affect their working efficiency. Equation (1) provides the relationship between the electromagnetic power and electromagnetic torque of a brushless DC motor^16^.

1 $$ P_{em} (t) = C(t)\Omega (t) = \sum\limits_{i = 1}^{m} {e_{i} (t)i_{i} (t)} $$

In Eq. (1), $C(t)$ is the electromagnetic torque. $\Omega$ serves as the rotational speed. $m$ represents the quantity of phases. The back electromotive force of the $i$-phase is represented by $e_{i} (t)$. $i_{i} (t)$ serves as the current of the $i$-th phase. When the motor reaches a stable state of operation, the electromagnetic power value remains constant, and the motor power equation at this time is described by Eq. (2).

2 $$ C\Omega = 2EI $$

In Eq. (2), $E$ is the stable back electromotive force. $I$ is the stable current. $C$ is the stable electromagnetic torque. According to Lenz's law, the back electromotive force of the motor can be derived by changing the coil flux. $\frac{\pi }{p}$ is now set as a pole distance for the motor rotor movement, and Eq. (3) describes the expression of the back electromotive force.

3 $$ E = \frac{n}{4}\frac{2\phi }{{\pi /p}}\Omega $$

In Eq. (3), $n$ serves as the quantity of coil turns. $p$ serves as the pole pair number. $\phi$ is the magnetic flux. Equation (4) describes the expression for the magnetic flux when the coil is in a magnetic field.

4 $$ \phi = B_{e} S_{p} $$

In Eq. (4), $B_{e}$ serves as the maximum magnetic flux density in the air gap. $S_{p}$ serves as the surface area of the magnetic pole. When the magnetic flux changes linearly with the rotor position, the expression of electromagnetic torque is described by Eq. (5).

5 $$ C = nIB_{e} \frac{{S_{e} }}{2\pi } $$

Equation (6) describes the air gap area.

6 $$ S_{e} = 2pS_{p} $$

In a radial brushless DC motor, Eq. (7) describes its air gap area.

7 $$ S_{e} = \pi D_{s} L_{m} $$

In Eq. (7), $D_{s}$ is the inner diameter of the stator, and $L_{m}$ is the stretching length of the motor. It derives the electromotive force on the ground of Eqs. (3), (4), and (7), and obtains the expression described by Eq. (8).

8 $$ E = \frac{n}{4}B_{e} D_{s} L_{m} \Omega $$

**Figure 2:**
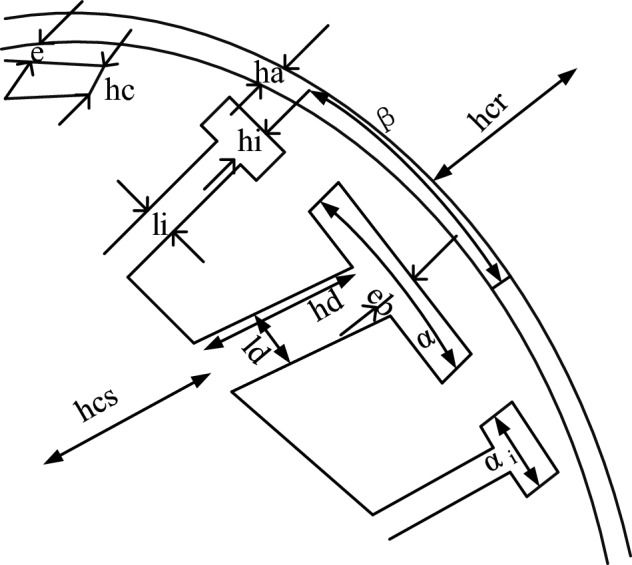
Schematic diagram of the key structure of brushless DC motor.

This study selects the key structural parameters of the brushless DC motor, as shown in Fig. 2.

Figure 2 is a schematic diagram of the key structure of a brushless DC motor. The key structural parameter formula of brushless DC motor is shown in Eq. (9).

9 $$ \left\{ {\begin{array}{*{20}l}    {S_{{enc}}  \approx hd[2\pi \left( {\frac{{D_{s} }}{2} - eb} \right) - \pi hd - N_{e} \left( {li + ld} \right)} \hfill  \\    {S_{{enc}} k_{r}  = \frac{3}{2}n\frac{I}{\sigma }} \hfill  \\    {D_{{ext}}  = D_{s}  + 2\left( {e + ha + hcr} \right)} \hfill  \\    {D_{{\text{int} }}  = D{}_{s} - 2\left( {eb + hd + hcs} \right)} \hfill  \\    {hc = \frac{{eb}}{{\cos \left( {\alpha /2} \right)}} - \frac{{D_{s} }}{2}\left[ {\frac{1}{{\cos \left( {\alpha /2} \right)}} - 1} \right]} \hfill  \\    {hi = \frac{{Ds}}{2}\left[ {1 - \cos \left( {\frac{{\alpha _{i} }}{2}} \right)} \right] + hc\cos \left( {\frac{{\alpha _{i} }}{2}} \right)} \hfill  \\   \end{array} } \right. $$

This study selects the key structural parameters of a brushless DC motor, with $S_{enc}$ as the total cross-section of the slot, the slot full ratio $k_{r}$ and $k_{r} < 1$, the current density in the conductor $\sigma$, $D_{ext}$ and $D_{{\text{int}}}$ as the outer diameter and inner diameter, and $N_{e}$ as the quantity of slots. Then it derives the height $h_{c}$ from the angle in the main tooth and the pole shoe, with the thickness of the middle tooth being $h_{i}$, and the height between the middle tooth and the end being the same as the height of the main tooth. Equation (10) describes the law of flux conservation in the main magnetic circuit of a motor.

10 $$ B_{d} Id = B_{e} \alpha \frac{{D_{s} }}{2} $$

In Eq. (10), $B_{d}$ is the magnetic induction intensity of the motor teeth. The magnetic flux from the permanent magnet of the motor passes through both sides of the rotor yoke, and Eq. (11) describes the conservation relationship of the magnetic flux.

11 $$ \frac{1}{2}B_{\alpha } \beta (\frac{{D_{s} }}{2} + e) = B_{cr} hcr $$

In Eq. (11), $B_{\alpha }$ is the magnetic induction intensity of the rotor yoke. $B_{\alpha }$ is the residual magnetism of the permanent magnet. Equation (12) describes the phase resistance.

12 $$ R_{ph} = \rho_{cu} (1 + \alpha_{cu} T_{cu} )\frac{n}{2}L_{ds} \frac{\delta }{I} $$

In Eq. (12), $\alpha_{cu}$ serves as the resistivity of the copper wire at 0 degrees Celsius. $\alpha_{cu}$ is the thermal coefficient and $\alpha_{cu} > 0$. $T_{cu}$ serves as the coil temperature, and the copper loss expression is derived according to Eq. (12), which is described by Eq. (13).

13 $$ R_{j} = 2R_{ph} I^{2} $$

The motor efficiency is described by Eq. (14).

14 $$ \eta = \frac{{C\Omega - P_{m} }}{{C\Omega + P_{j} + P_{f} }} $$

In Eq. (14), $P_{m}$ represents mechanical loss. This study proposes an optimization mathematical model for brushless DC motors on the ground of the selection of internal electromagnetic structural parameters and efficiency calculation theory. The model includes five optimization parameters and six constraint conditions. Among them, the optimization parameters are stator diameter, magnetic induction inside the teeth, winding current density, magnetic induction inside the air gap, and stator yoke magnetic induction. The constraints are the total mass of the motor, outer diameter, inner diameter, maximum phase current, slot height, and DC motor temperature. On this basis, this study considers that as the core component of the drive system, brushless DC motors can be optimized using mathematical methods such as sequential quadratic programming and direct search methods, which transform parameter issues into OPR. However, the computational efficiency is low and the optimization effect is not significant^17^. Thanks to the development of new intelligent biomimetic optimization algorithms recently, swarm intelligence algorithms is popular in many engineering OPR^18^. The improved JAYA algorithm is used to solve the optimal mathematical model of a brushless DC motor. The study utilizes the JAYA algorithm to both constrained and unconstrained OPR. As an algorithm with simple formulas and powerful heuristic characteristics, this algorithm can accelerate the CSP of the population through a single information exchange. Individual updates are only guided by the best and worst solutions^19^. However, due to the single way of information exchange, it is easy to cause the problem of missing population diversity, and it is easy to make the convergence state premature and fall into local optima^20^. So this study is on the ground of the JAYA algorithm and presents an improved JA for optimizing brushless DC motors.

The core idea of the JAYA algorithm is that for each candidate solution of a specific problem, the motion should approach the optimal solution while also adhering to the principle of the worst solution. Equation (15) depicts the traditional JAYA algorithm's process for updating the population.

15 $$ x_{i,j}{\prime} = x_{i,j} + r_{1} \cdot (x_{best,j} - \left| {x_{i,j} } \right|) - r_{2} \cdot (x_{worst,j} - \left| {x_{i,j} } \right|) $$

In Eq. (15), $r_{1}$ serves as the value of the $j$-th variable in the optimal solution. $x_{worst,j}$ is the value of the $j$-th variable in the worst-case solution. $r_{1}$ and $r_{2}$ represent two random numbers within the range of [0,1], respectively.

**Figure 3:**
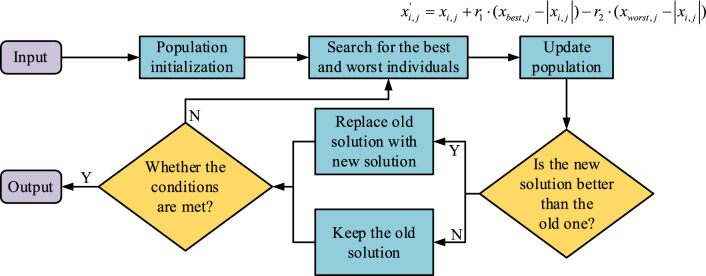
Flow of JAYA algorithm.

Figure 3 showcases the JAYA algorithm, which indicates that after initializing the population size and iteration times, then searching for optimal and worst individuals in the population. Next, it updates the population and compares the new and old solutions on the ground of the optimal individual found in the previous step. If the old solution is better than the new solution, the old solution is retained. Finally, it determines the obtained solution, and if it does not meet the conditions, it returns to the second step to search for the optimal and worst individuals in the population again.

The search direction of an individual depends on both the optimal solution and the worst solution, although the search method can enhance the search ability and CSP of the population. However, this study considers that while accelerating the CSP of the algorithm, it may also be accompanied by a decrease in population diversity. When encountering the current optimal solution, the convergence state is prone to falling into local optima. So this study introduces other experiential individual learning strategies to improve their local development ability while ensuring population diversity. Equation (16) describes this process.

16 $$x_{i,j}{\prime} = \left\{ \begin{gathered} x_{i,j} + r_{1} \cdot (x_{best,j} - \left| {x_{i,j} } \right|) + r_{2} \cdot (x_{m,j} - x_{n,j} ),{\kern 1pt} {\kern 1pt} {\kern 1pt} if{\kern 1pt} {\kern 1pt} {\kern 1pt} f(x_{m} ) < f(x_{n} ) \hfill \\ x_{i,j} + r_{1} \cdot (x_{best,j} - \left| {x_{i,j} } \right|) + r_{2} \cdot (x_{n,j} - x_{m,j} ),{\kern 1pt} otherwise{\kern 1pt} \hfill \\ \end{gathered} \right.  $$

In Eq. (16), $r_{1}$ serves as the value of the $j$-th variable in the optimal solution. $x_{m,j}$ and $x_{n,j}$ are the $j$-th dimensional variables of randomly selected individuals in the current population, and ensure $m \ne n \ne i$.

**Figure 4:**
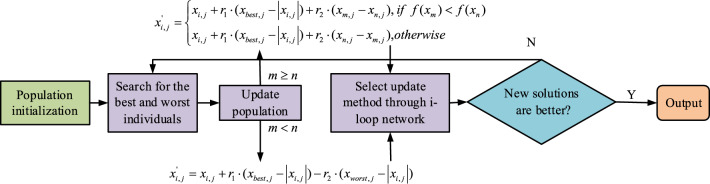
Flow of improved JAYA algorithm.

Figure 4 showcases the process of the improved JAYA algorithm, which indicates that after the improved JAYA algorithm initializes the population size, the algorithm operates on the objective function (OFU) of each individual to obtain the optimal and worst individuals. Then it enters a recurrent network and randomly selects population update formulas to maintain the balance of algorithm search and development capabilities. After each population update, it compares and determines the new solution with the old solution. For each determination, the cycles' quantity increases by one. When the number of cycles reaches the population size, the loop network will jump out and make a final decision on the current optimal solution. If the final condition is not met, it will return to search for the best and worst individuals again.

In order to ensure the optimization effect of brushless DC motors, research is conducted on the model using statistical analysis methods for data preprocessing, feature selection, model training, and model evaluation. The characteristics of statistical analysis methods mainly include scientificity, intuitiveness, and repeatability. Statistical analysis methods are based on mathematics and have a rigorous structure, requiring specific procedures and norms to be followed. When establishing a topic, proposing hypotheses, sampling, implementing specific methods, analyzing and interpreting data, and drawing conclusions, it is important to adhere to certain logic and standards. Statistical analysis methods collect data from real-life situations, which are presented through intuitive and simple quantitative numbers such as order and frequency, as well as concise charts. The processing of this data closely connects our research with the objective world, thereby revealing and understanding the essence and laws of the real world. Repeatability is an objective measure of the quality and level of research, and research conducted using statistical analysis methods is repeatable. From topic selection, sampling design, to data collection and processing, all can be repeated under the same conditions, and the results obtained from the study can be verified.

### Optimization of brushless DC motor based on adaptive JAYA algorithm

On the basis of the improved JAYA algorithm, this study considers that maintaining the balance between population diversity and improving CSP is a challenge. Population diversity can reflect the search performance of algorithms in different search areas within the search space when optimizing single objectives, but evolutionary algorithms generally have the problem of fast convergence leading to loss of population diversity^21^. When the algorithm searches for the optimal results in some regions, the convergence state will fall into local optima, making it challenging to break free and continue its search for global optima^22^. Introducing adaptive strategies is an effective method. Adaptive strategies can enable algorithms to have better development capabilities and search performance, allowing for better exploration of the solution space and the discovery of more optimal solutions^23^. So further improvements are made to the improved JAYA algorithm by introducing adaptive strategies to balance the algorithm's development ability and search performance^24,25^. The JAYA algorithm's CSP is slower than that of the PSO algorithm, and the minimum value of 30 runs is poor, indicating poor algorithm stability. This may be due to an imbalance between the algorithm's CSP and diversity during its design. Therefore, further improvements are necessary to enhance its performance and optimization ability. The algorithm utilizes an adaptive selection mechanism to adaptively select different learning strategies and balance the exploration and development ability of the algorithm. To achieve this, an adaptive weight strategy is introduced into the learning strategy of the basic JAYA algorithm. This strategy controls the degree to which each individual approaches the best solution and avoids the worst solution. On this basis, a hybrid learning strategy based on the optimal solution and other individual experiences is proposed. This strategy maintains algorithm diversity and improves local search ability. The chaotic elite search strategy is introduced to refine the optimal solution of each generation.

Specifically, on the ground of the introduction of a new social learning approach to enrich population diversity and avoid premature convergence, the adaptive selection mechanism structure enables individuals in the population to make adaptive choices from the original learning strategy and the newly introduced learning strategy.

In addition, this study combines the adaptive weight strategy with the learning strategy of the original JAYA algorithm, and uses a chaotic elite search strategy to refine the optimal solution for each iteration. Under ideal conditions, each individual in the population must closely follow the current optimal individual in the early stages of iteration, ultimately expediting the CSP towards the optimal potential region. When convergence reaches a later stage, individuals in the population should focus on the local area of the search to prevent premature convergence from missing the global optimal solution. Equation (17) describes the update formula of the algorithm after introducing an adaptive strategy.

17 $$ x_{i,j}{\prime} = x_{i,j} + w_{1} \cdot r_{1} \cdot \left( {x_{best,j} - \left| {x_{i,j} } \right|} \right) - w_{2} \cdot r_{2} \cdot \left( {x_{worst,j} - \left| {x_{i,j} } \right|} \right) $$

In Eq. (17), $r_{1}$ serves as the value of the $j$-th variable in the optimal solution. $x_{worst,j}$ is the value of the $j$-th variable in the worst-case solution. $r_{1}$ and $r_{2}$ represent two random quantities within the range of [0,1], respectively. Both $w_{1}$ and $w_{2}$ represent weight values. Equation (18) describes the adaptive adjustment process of two weight values as they change with the iterations' quantities.

18 $$ \left\{ \begin{gathered} w_{1} = \sin \left( {\frac{\pi t}{{2t_{\max } }} + \pi } \right) + 1 \hfill \\ w_{2} = \cos \left( {\frac{\pi t}{{2t_{\max } }} + \pi } \right) + 1 \hfill \\ \end{gathered} \right. $$

In Eq. (18), $t$ represents the current quantity of iterations. $t_{\max }$ represents the iterations' maximum quantities. As the iterations' quantities increases, the trend of the value of $w_{1}$ decreases from 1 to 0, while the value of $w_{2}$ increases from 0 to 1. The two weight parameters can be automatically determined on the ground of the number of iterations, which can save parameter adjustment work^26^. So in the early stages of population evolution, the motion control of individuals in the population is influenced by the optimal solution obtained from the current iteration, to achieve rapid convergence at the early. At the later of population evolution, most individuals gather, which can reduce the distance between individuals and the optimal solution for avoiding the worst solution^27,28^. This method of local search in the clustering area can enhance the optimal solution while ensuring that the population can escape the local optimal dilemma.

In the traditional JAYA algorithm, the selection of candidate solutions is usually directly influenced by the optimal and worst solutions, which can enhance the CSP of the population, but also hinder population diversity^29–31^. This is because the selection method does not take into account the search experience of other individuals, resulting in a high degree of individual diversity but a low degree of population diversity. So this study proposes a new blended learning strategy. Specifically, this strategy first randomly selects individuals from the current population, and individuals use the current best solution as their evolutionary best strategy. Through this social learning approach, the speed of individual fusion is improved. Meanwhile, for strengthening the diversity of the population, each individual in the population also needs to be guided by other individuals. This way of mutual learning between individuals can strengthen the local development capability, which is beneficial for the population for breaking out of the local convergence dilemma and obtain the global search optimal solution.

**Figure 5:**
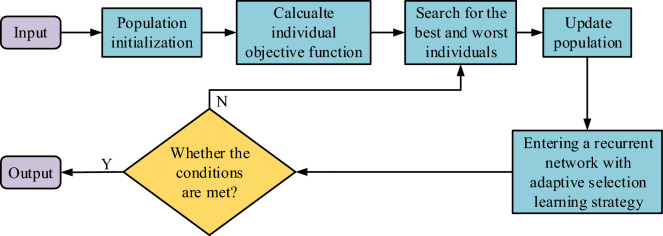
Flow of Adaptive JAYA Algorithm.

After introducing a blended learning strategy, it is necessary to construct an adaptive selection mechanism for each individual. Each individual can adaptively choose between two strategies, namely the introduction of adaptive weight strategy or hybrid learning strategy. This study uses the changes in fitness values of two adjacent generations of an individual to describe their current evolutionary process, to obtain the individual's selection probability for two learning strategies. So, on the basis of ensuring the individual's global search ability and local development ability, two different learning strategies are adaptively assigned according to the individual's evolutionary state. In the early stages of iteration, the individual's fitness value changes significantly, and the selection probability value during this period is small. At this point, the individual chooses a learning strategy with adaptive weights. As individuals progress to later stages, there is a gradual decrease in the change of fitness values, ultimately leading to an increased likelihood of selection. At this point, individuals choose a blended learning strategy to improve their local development ability. When an individual stops evolving or deteriorates in the process, it means that the population is trapped in a local optimal dilemma, and the selection probability value will be greater than or equal to 1. At this point, the study selects a hybrid learning strategy for the individual. For enhancing the algorithm's capability for jumping out of local optima, this study also introduces a chaotic elite search strategy. In the JAYA algorithm, the optimal solution for each iteration directly affects the evolutionary direction of other individuals. When the optimal solution of the previous generation exists in the local optimal region, it will guide other individuals to evolve towards the local optimal region, resulting in premature convergence. Therefore, relying on the random traversal characteristics of the chaotic elite search strategy, the quality of the optimal solution is further refined. Chaotic sequences enhance local search capabilities by causing strong perturbations to the optimal solution for each iteration. Figure 5 showcases the adaptive JAYA algorithm, which shows that after population initialization, the OFU of each individual is first calculated to search for the optimal and worst individuals in the population, and then enters the recurrent network. In a recurrent network, the current individual is judged: when the current individual is the optimal individual, the next iteration update is continued according to the chaotic elite local search strategy. When the current individual is not the optimal individual, its selection probability is determined. When the selection probability is greater than 1, update according to the hybrid learning strategy. When the selection probability is less than 1, it is updated according to the adaptive weight learning strategy. When the quantity of cycles is more excellent than or equal to the population size, the final decision is whether the updated optimal solution meets the conditions. If it does not, it returns to search for the optimal and worst individuals in the population again.

### Experimental results and analysis

For testing the improved JAYA algorithm for brushless DC motor OPR and the adaptive JAYA algorithm for brushless DC motor OPR, this study conducted experiments in a 64 bit Windows 10 PC environment with an Intel i5 9300H processor and 16 GB RAM size. To avoid accidental experimental results as much as possible, the number of independent runs for each benchmark function of the operation is set to 20. This study used the CEC2013 test function suite to analyze the performance of different algorithms.

### Performance experimental results and analysis of improved JAYA algorithm for optimization of brushless DC motors

For verifying the improved JAYA algorithm, this study selected the pre improved JAYA algorithm, the PSO, and Pigeon inspired Optimization (PIO) as comparative objects. The study tested it in an environment consisting of 10 items including multimodal and unimodal functions, where unimodal functions can evaluate the development capability, and multimodal functions can evaluate the exploration capability. F1-F3 is a unimodal function, F4-F8 is a multimodal function, and F9-F10 is a composite function. The characteristic of the F1 function is unimodal and separable. The characteristic of the F2 function is unimodal and separable, with varying sensitivities to variables. The characteristics of F3 function are multimodality, asymmetry, and a huge number of local optima. The characteristics of F4 function are multimodal, indivisible, and asymmetric. The characteristics of F5 function are multimodal, indivisible, asymmetric, continuous but only differentiable at a set of points. The characteristics of F6 function are multimodality, rotation, and asymmetry. The characteristics of the F7 function are multimodality, inseparability, asymmetry, and a large number of local optima. The characteristics of the F8 function are multimodal, indivisible, and asymmetric. The characteristics of F9 function are multimodal, inseparable, asymmetric, and different local optimal solutions have different properties. The characteristics of the F10 function are multimodal, indivisible, asymmetric, and different local optimal solutions have different properties. Table 2 provided the relevant parameter settings.

**Table 2:** Parameter settings of the algorithm.

Algorithm	Parameter settings
Improved JAYA	$NP$ = 100,$Nc$ = 20,000
JAYA	$NP$ = 100,$Nc$ = 20,000
PSO	$NP$ = 100,$Nc$ = 20,000,$w_{1}$ = [0,1],$c_{1}$ = 1.6,$c_{2}$ = 1.8
PIO	$NP$ = 100,$T_{1}$ = 12,000,$T_{2}$ = 8000,$R$ = 0.3
Parameter description	$NP$:Population size
	$Nc$:Iterations
	$w_{1}$:Inertia weight
	$c_{1}$,$c_{2}$:Learning factor
	$T_{1}$:Number of iterations for map and compass operations
	$T_{2}$:Number of iterations for landmark operations
	$R$:Map and compass factor

This study sets the dimension of all test functions to 20, the search range for all functions was [-100100], and the maximum number of evaluations per run was 500,000. The running environment was MATLAB. It selected the optimal value, worst case, average value, standard deviation, success rate, and rank as experimental indicators. Table 3 presented the operational results data of the improved JAYA algorithm and the original JAYA algorithm, which indicated that relative to the original JAYA algorithm, the improved JAYA algorithm possessed a lower rank average of 1.48 in unimodal function operations. This demonstrates stronger local development capabilities and better stability. The rank average of the original JAYA algorithm was 4.79, which was 3.31 higher than the improved JAYA algorithm, reflecting the effectiveness of other experiential individual learning strategies. In addition, in the operation of multimodal functions, the improved JAYA algorithm showed greater competitiveness, with a rank average of 1.34. It exhibits strong search ability in many local optima of multimodal functions, with a rank average of 3.63 lower than the original JAYA algorithm.

**Table 3:** Calculation results data of the improved JAYA algorithm and the original JAYA algorithm.

Algorithm index	Optimal value	Worst value	Average value	Standard deviation	Rank mean
Result of unimodal function operation					
Improved JAYA	0.00212	24.20	0.881	4.72	1.48
JAYA	27.8	28.6	27.4	0.49	4.79
Result of multimodal function operation					
Improved JAYA	0.285	0.114	0.00321	0.00511	1.34
JAYA	0.112	1.08	0.479	0.266	4.97

**Figure 6:**
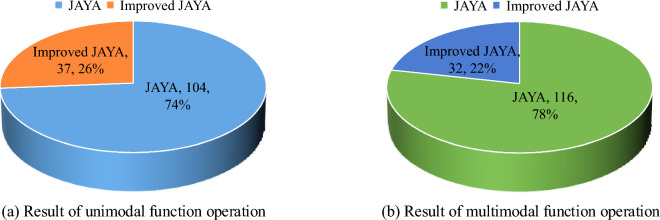
Rank Sum Comparison.

Figure 6 shows the comparison of the rank sum between the original JAYA algorithm and the improved JAYA algorithm in the operation of 20 unimodal and 20 multimodal functions. This indicated that the relevant rank sum during the unimodal function operation was 37, which was 67 lower than the original JAYA algorithm and about one-third of the original JAYA algorithm's rank sum. In the multimodal function operation, the rank sum of the improved JAYA algorithm was 32, which was 84 lower than the original JAYA algorithm and about a quarter of the rank sum of the original JAYA algorithm.

**Figure 7:**
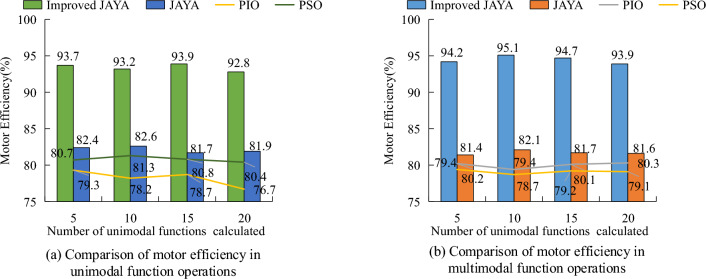
Comparison of motor efficiency of four algorithms.

To compare the relevant aspect of the improved JAYA algorithm with other algorithms used for OPR, its motor optimization efficiency was compared under 20 unimodal functions and 20 multimodal functions. Figure 7 showcases the efficiency comparison of the improved JAYA algorithm, pre improved JAYA algorithm, PSO algorithm, and pigeon swarm optimization (PISO) algorithm in the experiment. This showcased that the overall average efficiency of the improved JAYA algorithm was superior to the other three comparative algorithms. In the operation of 20 unimodal functions, the average efficiency of the improved JAYA algorithm was 93.4%, and the variation between the maximum average efficiency and the minimum average efficiency was within 1.1 percentage points. In contrast, the overall efficiency of the PISO algorithm and the original JAYA algorithm ranged from 80 to 83%, and the fluctuation range between the maximum and minimum efficiency values was greater than that of the improved JAYA algorithm. Compared to the improved JAYA algorithm, the average efficiency of the two algorithms was about 15 and 11 percentage points lower, respectively. In the operation of 20 multimodal functions, the average efficiency of the improved JAYA algorithm was 94.48%, and the variation between the maximum average efficiency and the minimum average efficiency was within 1.2 percentage points. Compared to other comparative algorithms, the improved JAYA algorithm had a more significant optimization effect.

**Figure 8:**
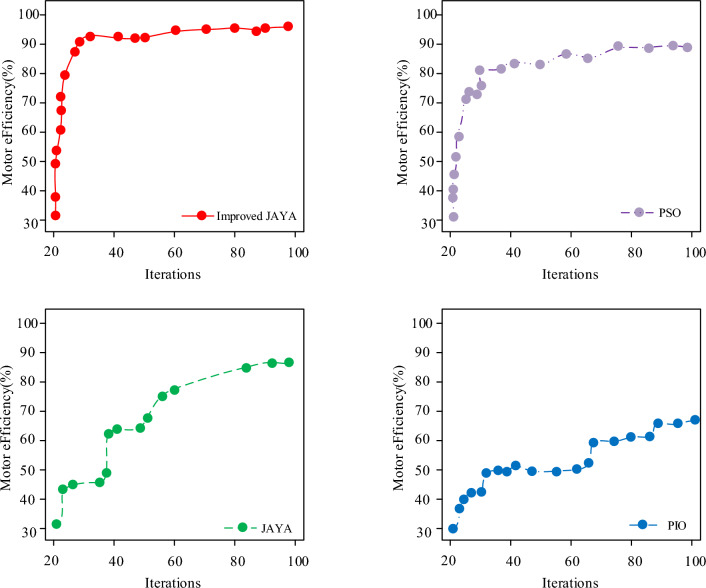
Convergence curves of four algorithms.

Figure 8 showcases a comparison of the convergence curves of the improved JAYA algorithm, the original JAYA algorithm, the PSO algorithm, and the PISO algorithm. This indicated that in the early stage of convergence, where the number of iterations ranged from 20 to 50, the original JAYA algorithm, PSO algorithm, and PISO algorithm inevitably fall into local optima. The improved JAYA algorithm has found the global optimum when the number of iterations reached around 40, and had faster CSP and accuracy. Although the efficiency of the pigeon swarm optimization algorithm improves with iteration, the final efficiency is lower than the improved JAYA algorithm. Although the PSO algorithm has good CSP, it may compete with local optimal solutions during the search process, leading to falling into local optimal solutions. Through evolution, the optimal solution is finally obtained in the 90th generation.

### Performance experimental results and analysis of adaptive JAYA algorithm for optimization of brushless DC motors

To verify the effectiveness of different strategies for algorithm improvement, three variants of JAYA algorithm were set as control algorithms. They were JAYA1 without adaptive weight strategy, JAYA2 without hybrid learning strategy, and JAYA3 without chaotic elite search strategy. In addition, when verifying the adaptive improved JAYA algorithm in dealing with motor OPR, the study selected the original JAYA algorithm, Brain Storm Optimization algorithm (BSO), and Differential Evolution (DE) algorithm as control algorithms. Table 4 showed the algorithm parameter settings.

**Table 4:** Parameter settings of the algorithm.

Algorithm	Parameter settings
Adaptive JAYA	$NP$ = 100,$Nc$ = 20,000
JAYA	$NP$ = 100,$Nc$ = 20,000
BSO	$NP$ = 100,$Nc$ = 20,000,$K$ = 3,$P_{5a}$ = 0.2,$P_{6b}$ = 0.8,$P_{6b3}$ = 0.4,$P_{6c}$ = 0.5
DE	$NP$ = 100,$Nc$ = 20,000,$CR$ = 0.9,$F$ = 0.5
Parameter description	$NP$:Population size
	$Nc$:Iterations
	$K$:Cluster size
	$P_{5a}$:Probability of directly updating the central cluster
	$P_{6b}$:The probability of selecting a clustered population
	$P_{6b3}$:Probability of selecting the selected cluster center
	$P_{6c}$:Probability of selecting two cluster population centers
	$CR$:Crossover probability
	$F$:Mutation probability

**Figure 9:**
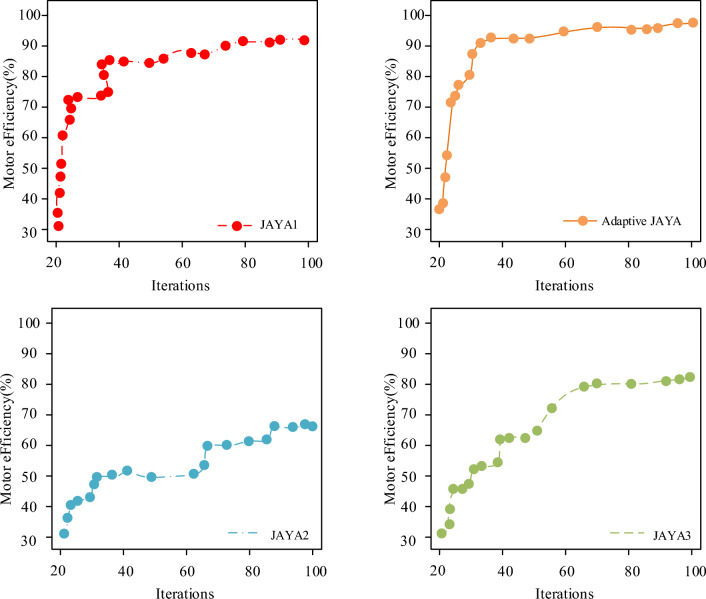
Dynamic performance comparison of different variant algorithms.

Figure 9 showcases the dynamic performance comparison of different variant algorithms during the iteration. Figure 8 showcased that during the iteration process, the adaptive JAYA algorithm converges faster than the other three comparative algorithms, had strong local development ability, and can achieve the highest efficiency. Compared to the JAYA2 and JAYA3 variants, although the CSP of the JAYA1 variant was faster, the motor efficiency obtained by its final convergence was still lower than that of the adaptive JAYA algorithm. The convergence accuracy of the three variants of the JAYA algorithm was lower than that of the adaptive JAYA algorithm that integrated the three learning strategies, which was effective in improving the JAYA algorithm by introducing an adaptive selection strategy. The results indicate that the adaptive JAYA algorithm performs poorly in solving complex combination functions. Additionally, other algorithms also exhibit suboptimal results in solving such functions, which may be attributed to their limited evolutionary iterations or global optimization capabilities.

**Figure 10:**
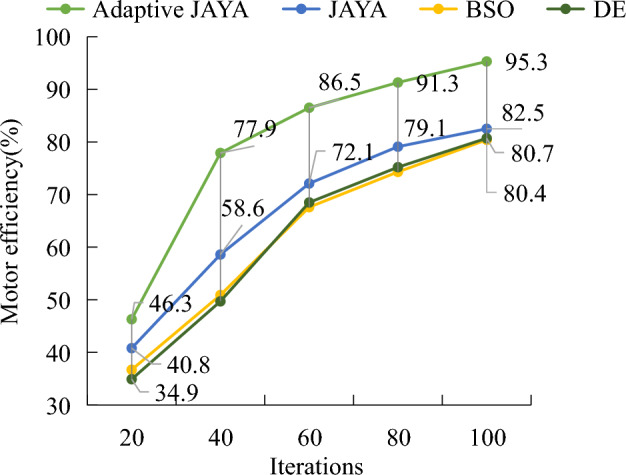
Comparison of motor efficiency changes under different algorithm optimizations.

Figure 10 showcases the comparison of motor efficiency changes between the adaptive JAYA algorithm and the original JAYA algorithm, brainstorming optimization algorithm, and DE algorithm during 100 iterations. Figure 9 showcased that with the quantity of iterations grows, the overall efficiency of the motor optimized by the four algorithms has been improved. The motor efficiency optimized by DE algorithm remained stable at around 80% when the number of iterations reaches 90, with a maximum efficiency of 80.7%. The brainstorming optimization algorithm stabilized at around 80% when the iterations' quantity reaches 90, with a maximum efficiency of 80.4%. The original JAYA algorithm remained stable at around 81% when the number of iterations reached 90, with a maximum efficiency of 82.5%. The adaptive JAYA algorithm was stable at around 93% when the iterations' quantity is 90, with a maximum efficiency of 95.3%. In contrast, the efficiency optimization of the adaptive JAYA algorithm had the largest increase, and the initial efficiency was higher than the other three comparison algorithms, at 46.3%, which was 5 to 12 percentage points higher than the other three comparison algorithms. The proposed hybrid learning strategy, along with the corresponding adaptive selection mechanism are indeed effective in improving the performance of the algorithm for solving brushless DC motor problems. However, it should be noted that the CSP of adaptive JAYA algorithm is faster than that of the basic JAYA algorithm, especially in the early stage. At the same time, chaotic local search is useful for refining the final solution, which enhances the algorithm's stability.

**Figure 11:**
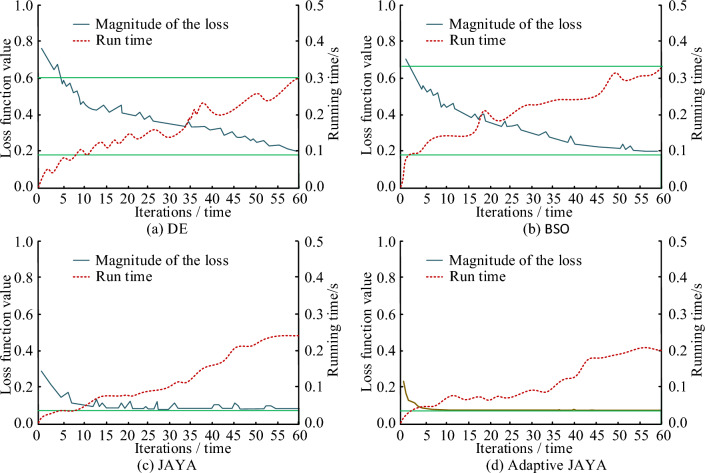
Error values and running time of four brushless DC motor solving algorithms in validation and testing sets.

In order to evaluate the convergence performance, running time, and accuracy of the adaptive JAYA algorithm, the error values and running time of four brushless DC motor solving algorithms in the validation and test sets are shown in Fig. 11a–d. In terms of runtime, the adaptive JAYA algorithm has shorter runtime under the same data size and iteration conditions, and when the number of iterations reaches about 40, the runtime will no longer change, while the stable runtime of other algorithms exceeds 50 times. Therefore, the convergence performance, running time, and accuracy of the adaptive JAYA algorithm have more obvious advantages over other brushless DC motor solving algorithms. As a whole, it can be seen that the error values of the four brushless DC motor solving algorithms in the training and validation sets gradually decrease with the increase of iteration times. The loss values in the validation set show periodic changes with the increase of iteration times. The changes in DE and BSO are more drastic compared to the other two algorithms, and the overall convergence speed is faster. At the same time, there is no gradient dispersion or explosion. Compared with other optimization algorithms, the adaptive JAYA algorithm has lower error values in both the training and validation sets, with stable loss values of 0.748 and 0.809, respectively. The DE algorithm has the highest error value in the training and validation sets, and when the number of iterations is 60, the loss value does not converge. The error values of the JAYA algorithm in the training and validation sets are second, followed by the BSO algorithm.

Table 5 showcases the optimal solutions of motor parameters obtained by four algorithms in multiple experiments. This indicated that compared to the theoretical parameter optimization values, the adaptive JAYA algorithm possessed the closest optimal solution for motor parameters to the theoretical parameter optimization values. This satisfied both the constraints of variables and the constraints of the model.

**Table 5:** Optimal solutions for motor parameters obtained from multiple experiments using different algorithms.

Algorithm parameter	Stator diameter	Intratooth magnetic induction	Winding current density	Magnetic induction in the air gap	Stator yoke magnetic induction
Theoretical parameter optimization values	201.2	1.8	2.044	0.65	0.89
Adaptive JAYA	201.5	1.8	2.049	0.63	0.91
JAYA	202.7	1.8	2.034	0.59	0.84
BSO	202.1	1.7	2.049	0.71	0.96
DE	203.4	1.7	2.051	0.62	0.84

**Figure 12:**
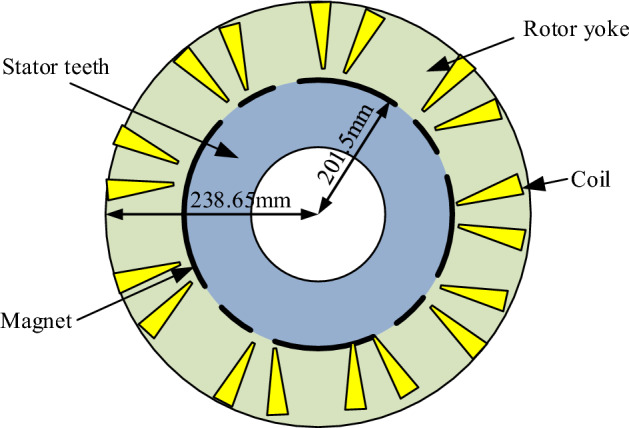
Cross section of the simulation model for brushless DC wheel motor based on the optimal solution of the adaptive JAYA algorithm.

Figure 12 showcases a cross-sectional view of the simulation model of a brushless DC wheel motor on the ground of the optimal solution of motor parameters obtained through multiple experiments using the adaptive JAYA algorithm. The stator diameter, tooth magnetic induction, winding current density, air gap magnetic induction, and stator yoke magnetic induction values were 201.5 mm, 1.8 T, 2.049 A/mm^2^, 0.63 T, and 0.91 T, respectively. All the parameters of the solution obtained by the algorithm not only meet the constraints of variables, but also meet the constraints of the model. This verifies the effectiveness of the algorithm.

In Fig. 12, the rotor of a brushless DC motor consists of a certain number of permanent magnets embedded on the surface of the iron core or embedded inside the iron core. Its structure generally adopted surface mounted magnetic poles. Surface mounted magnetic poles were tile like rare earth permanent magnets that are radially magnetized on the outer surface of the iron core. The stator was the stationary part of a brushless DC motor, consisting of an iron core, armature winding, and frame. The iron core of the stator usually consisted of laminated silicon steel sheets. The silicon steel sheets consisted of circular punched sheets with grooves, and the surface was insulated to reduce eddy current losses. The selection of slot numbers required a comprehensive consideration of the pole number of permanent magnets and the phase number of control circuits. Air gap referred to the gap between the stator and rotor.

**Figure 13:**
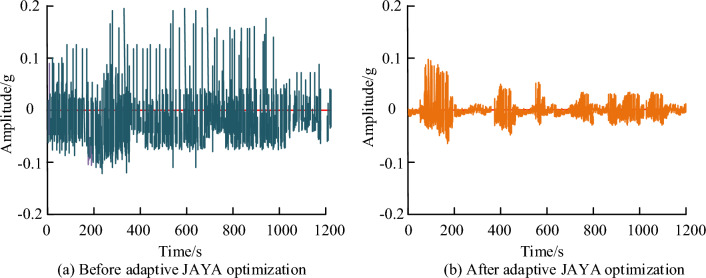
Vibration signals before and after wavelet coefficient threshold denoising.

Figure 13a and b show the vibration signals of the brushless DC wheel motor before and after optimization using the adaptive JAYA algorithm, respectively. The original signal contains a large amount of noise data. After processing the vibration signal using the adaptive JAYA algorithm, the changes in the original data can be largely preserved, and the obtained vibration signal tends to be smooth. Therefore, the adaptive JAYA algorithm can effectively remove noise data from the original signal and restore the feature data of the original data to the maximum extent. Based on the above analysis, the application of the adaptive JAYA algorithm in the optimization design of brushless DC wheel motors will significantly improve the stability and operational efficiency of the motor. Therefore, the adaptive JAYA algorithm has good adaptability in practical applications for optimizing parameters such as stator diameter, tooth magnetic induction, winding current density, air gap magnetic induction, and stator yoke magnetic induction.

**Figure 14:**
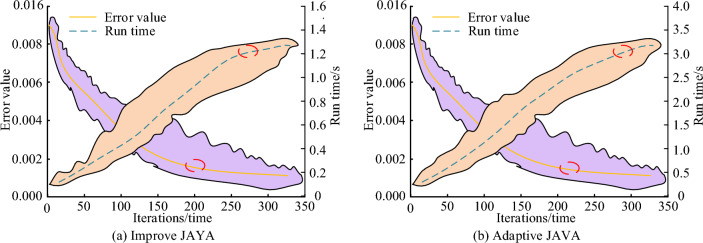
Convergence values and runtime of two algorithms.

To verify the effectiveness of the adaptive JAVA algorithm, this paper studies its effectiveness and an improved JAVA algorithm. Figure 14 shows the convergence value and running time of the two algorithms. Figure 14a shows that the error values of both algorithms decrease gradually with increasing iteration times. The convergence times are approximately 200 and 220 times, respectively. The stable error value of the fusion algorithm is 0.015, which is 29.36% higher than that of the single algorithm. Figure 14b showcases that the stable values of the two algorithms are 1.6 s and 1.7 s, respectively. This indicates that the iterative process of the fusion algorithm is not as fast as that of the single algorithm, but it is still within a reasonable numerical range, and the numerical difference is not big. Therefore, the fusion algorithm has advantages in both optimization time and error value. The improved JAYA algorithm has good performance and high efficiency in solving the optimization design problem of brushless DC motors, and is an effective optimization method to improve the operational efficiency of motors. Through comparative experiments with single objective benchmark functions, it has been shown that the improved JAYA algorithm has certain advantages in solving benchmark function problems. Solve the optimization design problem of brushless DC motors. Compared with existing algorithms, the adaptive JAYA algorithm has advantages in optimization performance, convergence speed, and stability. And the performance of different parts of the SAJAYA algorithm was tested, proving that all strategies are essential for improving algorithm performance. Meanwhile, a simulation model of a brushless DC motor was designed using the optimized parameters obtained from adaptive JAYA. Through single target benchmark testing experiments, it has been shown that compared with other algorithms, it exhibits certain advantages.

Finally, the study compares the design optimization methods of brushless DC motors using the latest literature cited techniques, with refs.^29–31^ as the comparison method. Table 6 shows the optimal solutions for five parameters obtained by different methods. Overall, the adaptive JAYA algorithm has the smallest difference from the actual theoretical values, while other design optimization methods have a significant difference from the actual theoretical values. In terms of the five parameters of stator diameter, tooth magnetic induction, winding current density, air gap magnetic induction, and stator yoke magnetic induction, the numerical difference in winding current density is the smallest, followed by the numerical differences in tooth magnetic induction, air gap magnetic induction, and stator yoke magnetic induction, and the numerical difference in stator diameter is the largest. Therefore, the adaptive JAYA algorithm has significant advantages.

**Table 6:** Five optimal solutions for parameters obtained by different methods.

Algorithm parameter	Stator diameter	Intratooth magnetic induction	Winding current density	Magnetic induction in the air gap	Stator yoke magnetic induction
Theoretical parameter optimization values	201.2	1.80	2.044	0.65	0.89
Adaptive JAYA	201.5	1.79	2.049	0.63	0.91
Reference^29^	201.9	1.79	2.038	0.60	0.85
Reference^30^	201.8	1.78	2.049	0.70	0.95
Reference^31^	202.0	1.78	2.046	0.63	0.85

**Figure 15:**
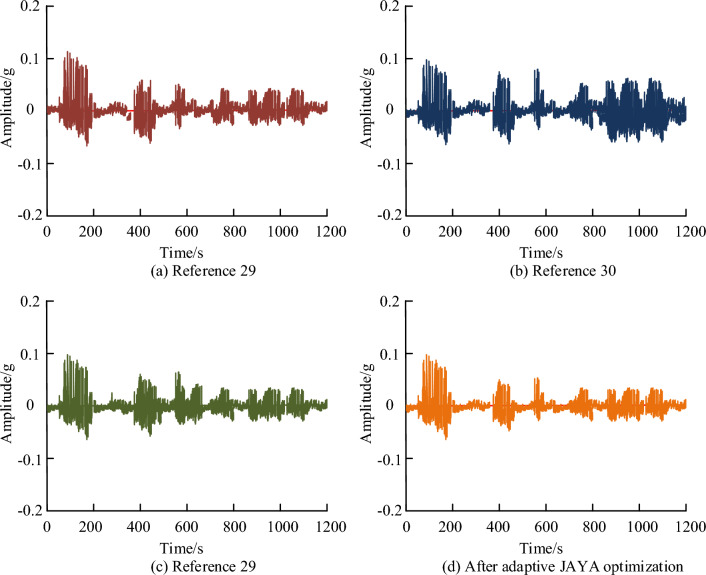
References^29–31^ and Adaptive JAYA Algorithm for Vibration Signals.

Figure 15a–d show the vibration signals of refs.^29–31^ and the adaptive JAYA algorithm, respectively. From the graph, it can be seen that the vibration signal fluctuation of the adaptive JAYA algorithm is the smallest, while the vibration signal fluctuation amplitude of other methods is larger, with the reduction rates of the maximum amplitude being 15.23%, 16.59%, and 26.35%, respectively. This may be due to the parameter settings not reaching the optimal level, resulting in a strong vibration signal. However, the vibration signals from refs.^29–31^ and the adaptive JAYA algorithm are both within the confidence interval. Through comprehensive analysis, it can be concluded that the adaptive JAYA algorithm has the best performance in solving the parameters of brushless DC motors, and has obvious advantages in practical applications.

## Discussion and conclusion

In OPR, the JAYA algorithm had a single way of exchanging information, which can easily lead to a lack of population diversity. This study was on the ground of the JAYA algorithm and proposes an improved JAYA algorithm to achieve optimization of brushless DC motors. Firstly, the study introduced an experiential learning strategy based on the JAYA algorithm to improve the population diversity of the algorithm. Subsequently, the study incorporated an adaptive algorithm based on individual evolution state on the basis of the first optimization. The improved algorithm for this study was ultimately formed. The experiment demonstrates that the improved JAYA algorithm had a lower rank average of 1.48 in unimodal function operations. This demonstrated stronger local development capabilities and better stability. The improved JAYA algorithm exhibits strong search ability in many local optima of multimodal functions. Its rank average was 3.63 lower than the original JAYA algorithm. The average efficiency of the improved JAYA algorithm was 94.48%, and the variation between the maximum and minimum average efficiency was within 1.2 percentage points. In contrast, the efficiency optimization of the adaptive JAYA algorithm had the largest increase, and the initial efficiency was higher than the other three comparison algorithms, at 46.3%, which was 5 to 12 percentage points higher than the other three comparison algorithms. The motor parameters obtained from the adaptive JAYA algorithm had a stator diameter of 201.5mm, intratooth magnetic induction of 1.8T, winding current density of 2.049 A/mm^2^, magnetic induction inside the air gap of 0.63T, and magnetic induction value of the stator yoke of 0.91T.The theoretical optimal parameter values for stator diameter, intratooth magnetic induction, winding current density, magnetic induction inside the air gap, and stator yoke magnetic induction were 201.2mm, 1.8T, 2.044 A/mm^2^, 0.65T, and 0.89T, respectively.

An improved JAYA algorithm is proposed. Based on the JAYA algorithm, this proposal suggests an empirical learning strategy to improve the diversity of the algorithm population. The strategy makes full use of the experience of other individuals to learn while avoiding the algorithm falling into local optimization. The experimental results show that the algorithm is competitive in solving the optimal design problem of brushless DC motors. In addition, an adaptive JAYA algorithm is proposed. The algorithm introduces an adaptive selection mechanism to adaptively select different learning strategies and balance the exploration and development ability of the algorithm. An adaptive weight strategy is introduced into the learning strategy of basic JAYA algorithm to control the degree that each individual approaches the best solution and avoids the worst solution. On the other hand, a hybrid learning strategy based on the optimal solution and other individual experiences is proposed. This strategy maintains the diversity of the algorithm population, improves the local search ability of the algorithm, and introduces a chaotic elite search strategy to refine the optimal solution of each generation. The experimental results show that the proposed algorithm has strong competitiveness in global optimization, convergence rate and algorithm stability, and at the same time, it improves the motor operation efficiency. At the same time, the performance and universality of the algorithm are tested. Yan Baked's algorithm is highly competitive in solving complex test functions. It has superior performance, including high precision and convergence, and good universality in other problems. Fractional order controller design via gazelle optimizer for efficient speed regulation of micromotors. The performance of a novel improved slim e-mould algorithm for direct current motor and automatic voltage regulator systems is also evaluated. This paper presents the design and application of a PID controller for regulating the speed of a DC motor. The controller is optimized using a hybrid Lévy flight distribution and Nelder–Mead algorithm.

The intelligent optimization algorithm given in this study brings a new solution to the design optimization problem of brushless DC motor. It overcomes the shortcomings of traditional calculation methods, such as easily falling into local optimization and difficult to deal with discrete variables, resulting in more accurate and efficient calculations. This paper studies the efficiency of the motor by optimizing the parameters of the motor and meeting the constraints of related parameters. The rate is maximized, thus reducing the production cost and improving the economic benefit. The optimal design of the motor only considers the single-objective efficiency maximization. However, in the practical application of brushless DC motor, it also needs to consider the minimum quality, so as to reduce the production cost and meet the important requirements of users. This can maximize the economic benefits. Therefore, the study of multi-objective optimization design of motor needs to establish a multi-objective optimization model to optimize, improve motor efficiency and reduce motor quality at the same time.

## Data Availability

The datasets used and/or analysed during the current study available from the corresponding author on reasonable request.
